# Medical News

**Published:** 1855-04

**Authors:** 


### MEDICAL NEWS.

We have been informed that Dr. G. W. Norris and Dr. Wm. Pepper have been elected from the Pennsylvania Hospital as delegates to the American Medical Association.

---

At a meeting of the Medical Board of the Philadelphia Hospital, held Feb. 24th, 1855, Drs. Henry H. Smith and J. L. Ludlow were chosen delegates to the American Medical Association, at their next Annual Meeting in Philadelphia. J. L. Ludlow,

*Secretary of Medical Board of Pa. Hospital.*

---

Mr. Editor,—Allow me, through the columns of the Examiner, to call the attention of the profession to the "Obstetrical Supporter," as modified and improved by W. S. Daniels, of New York. This light and simple apparatus is designed to make pressure upon the back of the parturient female, to furnish a support for her hands during the pains, and to keep her body and limbs in a proper position during the labor. It consists of a broad strap passing across the back and around the hips, with a pad for the back, and to which are attached at either end straps extending to the knees and feet. When properly adjusted, it gives that firm support so comfortable to the parturient female, furnishes something upon which to pull with her hands during the pains, and keeps her body and limbs in the position most favorable for the management of the case. The peculiarity of this instrument is, that as the female pulls upon the hand-straps during the pains, she at the same time increases the pressure upon the back, flexes the lower extremities, draws the trunk forward, and increases the contractile power of the abdominal muscles.

I have not yet had an opportunity of using this supporter, but from a careful examination of it, I consider it admirably adapted to fulfil the indications for which it was designed. JOHN WILTBANK, M. D.

Having examined the apparatus described by Dr. W., I concur in his opinion of its adaptation to favor parturition, especially during its latter stage. THEOPHILUS E. BEESLEY, M. D.

The above instrument may be seen at Mr. Gemrig's, in Eighth below Chestnut St., East side.

—

AMERICAN MEDICAL ASSOCIATION.—The Eighth Annual Meeting of the American Medical Association will be held in the city of Philadelphia, on Tuesday, May 1, 1855.

The Secretaries of all Societies and other bodies entitled to representation in the Association, are requested to forward to the undersigned correct lists of their respective delegations *as soon as they may be appointed;* and it is *earnestly* desired by the Committee of Arrangements that the appointments be made at as early a period as possible.

The following are extracts from Article 2d of the Constitution:—

"Each local Society shall have the privilege of sending one delegate for every ten of its regular resident members, and one for every additional fraction of more than half of this number. The faculty of every regularly constituted medical college, or chartered school of medicine, shall have the privilege of sending two delegates. The professional staff of every chartered or municipal hospital containing a hundred inmates or more, shall have the privilege of sending two delegates, and every other permanently organized medical institution of good standing shall have the privilege of sending one delegate.

"Delegates representing the medical staff of the United States army and navy shall be appointed by the Chiefs of Army and Navy Medical Bureaux.

"The number of delegates so appointed shall be four from the army medical officers, and an equal number from the navy medical officers."

The latter clause, in relation to delegates from the army and navy, was adopted as an amendment to the constitution at the meeting of the Association, held in New York, in May, 1853.

FRANCIS WEST, M. D.,
One of the Secretaries, 352 Chestnut St., Philadelphia.

The medical press of the United States is respectfully requested to copy the above.

[We sincerely hope that the above request will be attended to by the Secretaries of Societies, &c. We are informed that very few lists have, as yet, been forwarded.—ED.]

MEDICAL SOCIETY OF THE STATE OF PENNSYLVANIA.—The Annual Session of the Society will be held in Hollidaysburg on the last Wednesday (30th) of May, at 10 o'clock in the forenoon.

The Secretaries of the several County Societies will please forward the lists of delegates to either of the Secretaries.

D. FRANCIS CONDIE, Philadelphia,
HENRY CARPENTER, Lancaster,
*Secretaries.*

